# Supplementary material for: Pre-Columbian earth-builders settled along the entire southern rim of the Amazon
Source: Nat Commun. 2018 Mar 27;9:1125. doi: 10.1038/s41467-018-03510-7 (PMC5871619; doi:10.1038/s41467-018-03510-7)
Supplement: Supplementary file 1 — Supplementary Information(PDF 3083 kb) [file 41467_2018_3510_MOESM1_ESM.pdf]

Supplementary Information

“Pre-Columbian earth-builders settled along the entire southern rim of the Amazon” (De Souza et al.)

Supplementary Note 1

In order to contextualise the new sites discovered in the UTB, we propose the following architectural-functional categories for previously studied earthworks in the SRA.

'Ceremonial centres' are geometrically-patterned ditched enclosures, spatially restricted to the west of the SRA, mainly in the Brazilian state of Acre, where they are known as 'geoglyphs'[1,2]. The forests of the state of Acre were previously thought to be pristine. However, large-scale deforestation after the 1980s revealed over 500 geoglyphs in an area of ca. 20,000 km². They are geometrically perfect and symmetrical square and circular enclosures, eventually including other shapes like hexagons and octagons, delimited by contiguous ditches and banks. Noticeable for their symmetry, the architecture of geoglyph sites can be complex, juxtaposing square and circular ditches, walled enclosures, mounds and causeways, potentially reflecting a long history of construction and remodelling. The ditches are on average 11 m wide and up to 4 m deep, with external embankments up to 2 m high. The enclosures generally surround an area of 1-3 ha, although larger sites exist. Avenues, delineated by low banks, frequently connect the separate enclosures and link them to a network of streams carved in the upland soils. They are located on the edges of plateaus 180-230 meters above sea level (masl) with good views of the surroundings, ca. 1.5-8 km from navigable river courses[3,4]. Paleocological work indicates they were built on bamboo-dominated forests[5] as is predicted comparing the distribution of modern bamboo (*Guadua* sp.) forest and archaeological sites in the region[6]. The low ceramic density, presence of votive deposits inside the ditches, and the lack of Anthropogenic Dark Earth (ADE) associated with the enclosures has led to their interpretation as public spaces for repeated gatherings and communal feasting, rather than as permanent habitations[2,4,7].

'Fortified settlements' are present across the whole SRA, from Acre to the Upper Xingu, 1500 km to the east. In the Llanos de Moxos region, Bolivia, they are known as *zanjas* or ring ditches. These sites can in general be recognised as irregularly-shaped,

asymmetrical enclosures. Unlike the previous category, encircling an area with a ditch appears to have been more important than achieving geometrical perfection. They can be roughly circular, elliptical, D-shaped or completely irregular. Ditches are up to 10 m wide and up to 4 m deep, and the enclosed areas cover on average 1-5 ha, with some larger sites exceeding 10-12 ha[8-10]. The closer proximity to water sets this type of site apart from the ceremonial geometric enclosures, as they are located in small plateaus overlooking rivers, and sometimes next to river margins. Paleoecological work indicates that ring ditches that are located in what is today the *terra firme* forest on the pre-Cambrian shield of Baures, Bolivia, were built when this region was savannah[11]. The elevated position in the landscape, presence of ADE, house floors, domestic debris and urn burials inside the enclosures confirms their use as fortified settlements[8,10,12]. Although archaeological evidence of palisades associated with the ditches is still lacking, the sites are likely to correspond to palisaded villages described in colonial accounts[9,12,13]. The habitation nature of most sites is also confirmed by our new data from the UTB, where many enclosures contained ADE and high density of ceramics. Nevertheless, many of the ditched enclosures could have fulfilled other functions, from burial grounds to water management systems, and variability in their function will become better understood as more fieldwork is carried out. In Acre, geometrical and irregular ditched enclosures appear in the same regions, sometimes as part of the same site. In the Llanos de Moxos, they are part of compounds, with adjoined enclosures and multiple concentric ditches. Some are associated with what appear to be hydraulic earthworks like canals, the complexity of which only recently has been documented through LiDAR survey[8,12]. In those compounds, a network of ditches connects the different enclosures to each other and to streams and rivers. Finally, along the headwaters of the Xingu River, Heckenberger et al.[14,15] have documented dozens of settlements, fortified by ditches and connected by a regional network of roads, in a “galactic” system of regional polities extending over 20,000 km^2. The Upper Xingu sites can be considered a unique regional development, distinguished by the large settlement size (20-50 ha) and network of roads.

‘Mounded ring villages’ consist of circles of mounds around a central plaza from where roads emanate in all directions. They are fundamentally distinct from the ditched enclosures in terms of architecture, but have been included due to their widespread occurrence and close spatial association with the previous site types. They are reminiscent of the Upper Xingu settlements in the pattern of roads radiating from a central plaza, but are distinguished by their mounded architecture and considerably smaller size (typically 120-160 m across, although the roads tend to extend for a few hundred kilometres more). Excavations at the mounds reveal clear occupation strata with domestic features and adjacent middens, confirming their nature as settlements[2,10,16]. In Acre and the adjacent Bolivian region of Riberalta, mounded ring villages appear next to ditched enclosures or inside them. Thus, their landscape placement is the same as the geometrical enclosures, on small plateaus at a distance of 1.5-8 km from navigable rivers. Ethnographic and archaeological ring villages, where houses are arranged in a circle around a ceremonial plaza, are common among central Brazilian groups[17,18]. In the SRA, ring villages are the typical settlement pattern of Arawak speakers[19-21].

Supplementary Tables

Supplementary Table 1. Radiocarbon dates for ceremonial centres (geoglyphs) in the SRA.

Site	^{14}C BP	Laboratory number	Reference
Balneario Quinauá	1565 ± 35	Ua-37263	Saunaluoma and Schaan [7]
Balneario Quinauá	1570 ± 35	Ua-37262	Saunaluoma and Schaan [7]
Balneario Quinauá	1585 ± 30	Ua-37260	Saunaluoma and Schaan [7]
Balneario Quinauá	1760 ± 35	Ua-37261	Saunaluoma and Schaan [7]
Fazenda Atlântica	1855 ± 30	Ua-37252	Saunaluoma and Schaan [7]
Fazenda Atlântica	1905 ± 35	Ua-37251	Saunaluoma and Schaan [7]
Fazenda Atlântica	2110 ± 35	Ua-37253	Saunaluoma and Schaan [7]
Fazenda Colorada	1340 ± 35	Ua-37236	Schaan, et al. [2]
Fazenda Colorada	1275 ± 30	Ua-37255	Schaan, et al. [2]
Fazenda Colorada	1865 ± 65	Ua-37235	Schaan, et al. [2]
Fazenda Colorada	1820 ± 30	Ua-37256	Schaan, et al. [2]
Fazenda Colorada	1775 ± 35	Ua-37567	Schaan, et al. [2]
Jacó Sá	1205 ± 30	Ua-37258	Schaan, et al. [2]
Jacó Sá	1195 ± 30	Ua-37257	Schaan, et al. [2]
Jacó Sá	1485 ± 35	Ua-37259	Schaan, et al. [2]
JK	1710 ± 30	Beta-294309	Saunaluoma and Schaan [7]
JK	1830 ± 30	Beta-294310	Saunaluoma and Schaan [7]
Ramal do Capatará	1850 ± 40	Beta-288232	Saunaluoma and Schaan [7]
Ramal do Capatará	1990 ± 30	Beta-288233	Saunaluoma and Schaan [7]
Ramal do Capatará	3310 ± 40	Beta-288234	Saunaluoma and Schaan [7]
Severino Calazans	3990 ± 40	Ua-37237	Schaan, et al. [2]
Severino Calazans	2915 ± 35	Ua-37238	Schaan, et al. [2]
Severino Calazans	2275 ± 35	Ua-37265	Schaan, et al. [2]
Severino Calazans	2050 ± 35	Ua-37264	Schaan, et al. [2]

Supplementary Table 2. Radiocarbon dates for fortified villages (Bolivian ring ditches and sites in the Upper Xingu) in the SRA.

Site	^{14}C BP	Laboratory number	Reference
Aliança	1655 ± 65	N/A	Simões [22]
Bella Vista-1	568 ± 43	Erl-6560	Prümers [23]
Bella Vista-1	634 ± 44	Erl-6561	Prümers [23]
Bella Vista-1	726 ± 41	Erl-6558	Prümers [23]
Bella Vista-2	607 ± 28	KIA-38833	Prümers [23]
Bella Vista-2	775 ± 25	KIA-48489	Prümers [23]
Bella Vista-2	782 ± 27	KIA-38831	Prümers [23]
Bella Vista-2	783 ± 25	KIA-48488	Prümers [23]
Candelaria	1700 ± 40	Ua-24928	Saunaluoma [10]
Chacra Teleria	1940 ± 40	Ua-24931	Saunaluoma [10]
El Círculo	1790 ± 75	Hela-570	Saunaluoma [10]
Estancia Giese	1815 ± 45	Hela-708	Saunaluoma [10]
Estancia Giese	1695 ± 40	Hela-709	Saunaluoma [10]
Estancia Girasol	475 ± 35	Ua-24929	Saunaluoma [10]
Jasiaquiri	444 ± 25	KIA-48486	Prümers [23]
Jasiaquiri	500 ± 25	KIA-48484	Prümers [23]
Jasiaquiri	596 ± 25	KIA-48487	Prümers [23]
Jasiaquiri	610 ± 25	KIA-48482	Prümers [23]
Laranjeira	585 ± 55	N/A	Simões [22]
Las Palmeras	1850 ± 40	Ua-24930	Saunaluoma [10]
Militão	2465 ± 55	N/A	Simões [22]
MT-FX-05	670 ± 60	Beta-177724	Heckenberger, et al. [14]
MT-FX-06	440 ± 60	Beta-176135	Heckenberger, et al. [14]
MT-FX-06	710 ± 50	Beta-176136	Heckenberger, et al. [14]
MT-FX-06	340 ± 60	Beta-176137	Heckenberger, et al. [14]
MT-FX-06	590 ± 60	Beta-176139	Heckenberger, et al. [14]
MT-FX-06	530 ± 60	Beta-176140	Heckenberger, et al. [14]
MT-FX-06	1030 ± 60	Beta-176141	Heckenberger, et al. [14]
MT-FX-06	700 ± 50	Beta-194840	Heckenberger, et al. [14]
MT-FX-06	720 ± 40	Beta-194841	Heckenberger, et al. [14]
MT-FX-06	750 ± 40	Beta-194843	Heckenberger, et al. [14]
MT-FX-06	180 ± 60	Beta-72260	Heckenberger, et al. [14]
MT-FX-06	1000 ± 70	Beta-72261	Heckenberger, et al. [14]
MT-FX-06	440 ± 70	Beta-72262	Heckenberger, et al. [14]
MT-FX-06	700 ± 70	Beta-78979	Heckenberger, et al. [14]
MT-FX-06	360 ± 70	Beta-81301	Heckenberger, et al. [14]
MT-FX-06	1370 ± 60	Beta-176143	Heckenberger, et al. [14]
MT-FX-06	690 ± 60	Beta-177724	Heckenberger, et al. [14]
MT-FX-06	1810 ± 40	Beta-194844	Heckenberger, et al. [14]
MT-FX-06	2110 ± 40	Beta-176138	Heckenberger, et al. [14]
MT-FX-11	900 ± 60	Beta-72262	Heckenberger, et al. [14]
MT-FX-11	440 ± 70	Beta-72263	Heckenberger, et al. [14]
MT-FX-12	190 ± 60	Beta-72264	Heckenberger, et al. [14]
MT-FX-13	890 ± 40	Beta-197515	Heckenberger, et al. [14]
MT-FX-13	930 ± 50	Beta-197516	Heckenberger, et al. [14]
MT-FX-13	690 ± 60	Beta-88362	Heckenberger, et al. [14]
MT-FX-13	910 ± 80	Beta-88363	Heckenberger, et al. [14]
MT-FX-13	1160 ± 80	Beta-197517	Heckenberger, et al. [14]
MT-FX-14	440 ± 40	N/A	Heckenberger, et al. [14]
MT-FX-14	440 ± 50	N/A	Heckenberger, et al. [14]
MT-FX-15	340 ± 50	N/A	Heckenberger, et al. [14]
Pimenteiras	240 ± 40	N/A	Simões [22]
Rolim de Moura	195 ± 45	N/A	Simões [22]
Tumichucua	1905 ± 40	Hela-702	Saunaluoma [10]
Tumichucua	2045 ± 65	Ua-24932	Saunaluoma [10]

Supplementary Table 3. Radiocarbon dates for mounded ring villages in the SRA.

Site	^{14}C BP	Laboratory number	Reference
El Círculo	600 ± 60	Hela-4585	Saunaluoma [10]
El Círculo	680 ± 30	Poz-9523	Saunaluoma [10]
El Círculo	715 ± 30	Poz-9426	Saunaluoma [10]
El Círculo	650 ± 30	Poz-9524	Saunaluoma [10]
El Círculo	660 ± 30	Poz-9427	Saunaluoma [10]
El Círculo	685 ± 30	Poz-9428	Saunaluoma [10]
El Círculo	645 ± 30	Poz-9429	Saunaluoma [10]
Fazenda Colorada	750 ± 35	Hela-616	Schaan, et al. [2]
Las Palmeras	285 ± 35	Ua-24076	Saunaluoma [10]
Sol de Campinas	440 ± 30	Beta-408412	Neves, et al. [16]
Sol de Campinas	530 ± 30	Beta-408410	Neves, et al. [16]
Sol de Campinas	660 ± 30	Beta-408409	Neves, et al. [16]
Sol de Campinas	730 ± 30	Beta-408407	Neves, et al. [16]
Sol de Campinas	960 ± 30	Beta-408408	Neves, et al. [16]

Supplementary Table 4. Archaeological sites identified in the Upper Tapajós Basin. Population estimates were calculated based on the linear equation described in Curet [24].

Site	Structure	Type	Latitude	Longitude	Area (ha)	Potential population
Mt01	I	circular enclosure	-57.9452	-9.4071	1.61	239
Mt02	I	hexagonal enclosure	-57.8872	-9.8711	3.18	454
Mt03	I	circular enclosure	-57.8772	-9.7048	5.11	719
Mt04	I	enclosure	-57.8212	-9.8132	2.41	349
Mt05	I	circular enclosure	-57.7557	-9.8221	1.77	262
Mt06	I	circular enclosure	-58.2325	-9.4150	1.82	268
Mt06	II	enclosure	-58.2326	-9.4149		
Mt07	I	circular enclosure	-59.3228	-9.3501	9.88	1373
Mt07	II	circular enclosure	-59.3208	-9.3512		
Mt07	III	causeway	-59.3146	-9.3476		
Mt08	I	circular enclosure	-59.2340	-9.4506	0.9	143
Mt09	I	circular enclosure	-58.6019	-9.9833	0.79	127
Mt10	I	hexagonal enclosure	-58.4585	-10.0984	1.75	258
Mt11	I	circular enclosure	-58.6438	-10.3111	2.07	302
Mt12	I	hexagonal enclosure	-57.3703	-9.4245	3.47	494
Mt13	I	circular enclosure	-58.4414	-10.7534	0.13	37
Mt14	I	hexagonal enclosure	-59.6343	-10.0918	6.88	962
Mt16	I	circular enclosure	-58.5072	-10.3604	0.16	41
Mt17	I	hexagonal enclosure	-57.9857	-9.7611	2.04	298
Mt18	I	enclosure	-57.8512	-9.4176	4.53	639
Mt19	I	circular enclosure	-57.8374	-9.5936	0.1	33
Mt20	I	enclosure	-57.7716	-9.8721	1.01	157
Mt21	I	circular enclosure	-59.0036	-9.4852	0.48	85
Mt23	I	circular enclosure	-55.2413	-9.9197	1.59	236
Mt24	I	causeway	-55.5070	-9.6730		
Mt25/26	I	circular enclosure	-58.5124	-9.9246	0.29	58
Mt25/26	II	circular enclosure	-58.5100	-9.9231		
Mt27	I	circular enclosure	-59.1692	-9.4302	0.45	81
Mt27	II	circular enclosure	-59.1690	-9.4304		
Mt28	I	circular enclosure	-59.2405	-9.3650	0.49	87
Mt29	I	circular enclosure	-59.3187	-9.2897	0.77	125
Mt29	II	causeway	-59.3240	-9.2909		
Mt30	I	mounded village	-59.3287	-9.4957	0.77	125
Mt-31	I	hexagonal enclosure	-60.0786	-9.9671	2.11	308
Mt-32	I	enclosure	-57.9185	-9.7542	2.63	379
Mt-33	I	circular enclosure	-58.5755	-10.0513	0.21	48
Mt-34	I	circular enclosure	-58.5713	-10.1108	0.06	28
Mt-35/36	I	enclosure	-58.5775	-10.0298	0.24	73
Mt-35/36	II	circular enclosure	-58.5766	-10.0297		
Mt-37	I	circular enclosure	-58.5424	-10.2691	0.19	45
NN	I	enclosure	-59.0066	-9.4575	1.54	230
Z-Mt01	I	circular enclosure	-58.1495	-9.7471	4.76	671
Z-Mt01	II	enclosure	-58.1493	-9.7476		

Z-Mt01	III	circular enclosure	-58.1500	-9.7489		
Z-Mt01	IV	circular enclosure	-58.1499	-9.7494		
Z-Mt02	I	circular enclosure	-58.1425	-10.1667	5.27	741
Z-Mt03	I	hexagonal enclosure	-57.7843	-9.5961	3.43	489
Z-Mt04	I	hexagonal enclosure	-57.6745	-9.8635	18.79	2594
Z-Mt04	II	enclosure	-57.6760	-9.8654		
Z-Mt05	I	circular enclosure	-58.3389	-9.3335	3.41	486
Z-Mt05	II	circular enclosure	-58.3391	-9.3329		
Z-Mt06	I	enclosure	-58.2823	-9.4370	3.6	512
Z-Mt07	I	circular enclosure	-58.1723	-9.4871	1.15	177
Z-Mt08	I	hexagonal enclosure	-58.1557	-9.4418	1.31	198
Z-Mt09/10	I	circular enclosure	-58.8590	-9.2415	0.59	100
Z-Mt09/10	II	circular enclosure	-58.8576	-9.2446		
Z-Mt09/10	III	circular enclosure	-58.8564	-9.2429		
Z-Mt09/10	IV	circular enclosure	-58.8565	-9.2427		
Z-Mt11	I	circular enclosure	-58.9347	-9.2116	0.35	67
Z-Mt11	II	circular enclosure	-58.9350	-9.2115		
Z-Mt12	I	enclosure	-59.0231	-9.0253	3.34	477
Z-Mt12	II	circular enclosure	-59.0221	-9.0253		
Z-Mt13	I	circular enclosure	-59.0994	-9.0478	0.73	119
Z-Mt14	I	circular enclosure	-59.0988	-9.3029	0.14	38
Z-Mt14	II	causeway	-59.1009	-9.2996		
Z-Mt14	III	causeway	-59.0970	-9.3012		
Z-Mt15	I	circular enclosure	-59.0958	-9.3129	0.04	25
Z-Mt16	I	circular enclosure	-59.1055	-9.0507	0.37	70
Z-Mt16	II	circular enclosure	-59.1072	-9.0509		
Z-Mt16	III	circular enclosure	-59.1049	-9.0497		
Z-Mt19	I	circular enclosure	-58.4527	-9.9663	0.14	38
Z-Mt20	I	circular enclosure	-58.3285	-10.3927	0.1	32
Z-Mt21	I	enclosure	-57.4240	-9.1798	8.92	1241
Z-Mt22	I	circular enclosure	-59.7402	-9.3220	0.15	39
Z-Mt23	I	circular enclosure	-59.7501	-9.4271	1.27	193
Z-Mt24/25	I	circular enclosure	-59.7943	-9.5016	2.12	309
Z-Mt24/25	II	enclosure	-59.7980	-9.5016		
Z-Mt25/26	II	enclosure	-59.8014	-9.4867	0.51	88
Z-Mt26	I	circular enclosure	-59.8006	-9.4867	0.82	131
Z-Mt27	I	circular enclosure	-59.8122	-9.5065	2.44	353
Z-Mt28	I	hexagonal enclosure	-57.1729	-9.7239	4.99	703
Z-Mt29	I	circular enclosure	-57.9733	-9.7970	0.11	34
Z-Mt30	I	hexagonal enclosure	-57.9374	-10.1606	1.52	227
Z-Mt31	I	enclosure	-57.7010	-9.2278	3.37	480
Z-Mt32	I	hexagonal enclosure	-57.6511	-9.6583	1.21	185
Z-Mt33	I	circular enclosure	-58.9042	-9.2813	0.25	53
Z-Mt33	II	enclosure	-58.9044	-9.2816		
Z-Mt34	I	mounded village	-59.2797	-9.4404		
Z-Mt35	I	circular enclosure	-58.2266	-9.5330	0.12	36
Z-Mt36	I	enclosure	-59.7847	-9.9606	1.17	180
Z-Mt37	I	circular enclosure	-59.8073	-9.8468	0.4	74
Z-Mt38	I	circular enclosure	-59.0461	-9.5301	0.37	69
Z-Mt38	II	circular enclosure	-59.0465	-9.5302		
Z-Mt39	I	circular enclosure	-60.2005	-9.6139	0.31	62
Z-Mt40	I	circular enclosure	-58.0065	-9.5131	0.14	39

Z-Mt41	I	enclosure	-58.427	-10.6544	4.71	665
Z-Mt42	I	circular enclosure	-59.0987	-9.3028	0.13	37
Z-Mt43	I	circular enclosure	-58.8564	-9.243	0.46	141
Z-Mt43	II	circular enclosure	-58.8565	-9.2426		
Z-Mt43	III	circular enclosure	-58.859	-9.2415		
Z-Mt43	IV	circular enclosure	-58.8575	-9.2446		
Z-Mt44	I	circular enclosure	-58.1381	-9.2874	0.07	29
Z-Mt45	I	enclosure	-59.0954	-9.6312	0.3	60
Z-Mt46	I	enclosure	-58.8959	-9.3249	0.19	46

Supplementary Table 5. Variables entered as predictors in the MaxEnt model. Variables in bold are those with the five highest percent contribution and/or permutation importance, whereas variables in red are highly correlated with others of easier interpretation and have not been included in the final model.

Bioclimatic	Bio1 = Annual Mean Temperature (degrees Celsius * 10)
	Bio2 = Mean Diurnal Range (Mean of monthly (max temp - min temp))
	Bio3 = Isothermality (Bio2/Bio7) (* 100)
	**Bio4 = Temperature Seasonality (standard deviation * 100)**
	**Bio5 = Max Temperature of Warmest Month (degrees Celsius * 10)**
	Bio6 = Min Temperature of Coldest Month (degrees Celsius * 10)
	**Bio7 = Temperature Annual Range (Bio5-Bio6)**
	Bio8 = Mean Temperature of Wettest Quarter (degrees Celsius * 10)
	Bio9 = Mean Temperature of Driest Quarter (degrees Celsius * 10)
	Bio10 = Mean Temperature of Warmest Quarter (degrees Celsius * 10)
	Bio11 = Mean Temperature of Coldest Quarter (degrees Celsius * 10)
	Bio12 = Annual Precipitation (mm)
	Bio13 = Precipitation of Wettest Month (mm)
	Bio14 = Precipitation of Driest Month (mm)
	Bio15 = Precipitation Seasonality (Coefficient of Variation)
	Bio16 = Precipitation of Wettest Quarter (mm)
	**Bio17 = Precipitation of Driest Quarter (mm)**
	Bio18 = Precipitation of Warmest Quarter (mm)
	**Bio19 = Precipitation of Coldest Quarter (mm)**
	Gravel content (%vol)
	**Sand fraction (% wt)**
	Silt fraction (%wt)
	Clay fraction (%wt)
	Reference bulk density (kg/dm3)
	Bulk density (kg/dm3)
	Organic carbon (%weight)
	pH (-log(H^+))
	**Cation exchange capacity (clay) (cmol/kg)**
	Cation exchange capacity (soil) (cmol/kg)
	Base saturation (%)
	Total exchangeable bases (cmol/kg)
	Calcium carbonate (%weight)
	Gypsum (%weight)
	Sodicity (ESP) (%)
	Salinity (ECe) (dS/m)
Terrain	**Elevation (m)**
	Slope (degrees)
	Terrain ruggedness index
	Topographic position index
	Distance to rivers (m)

Supplementary Figures

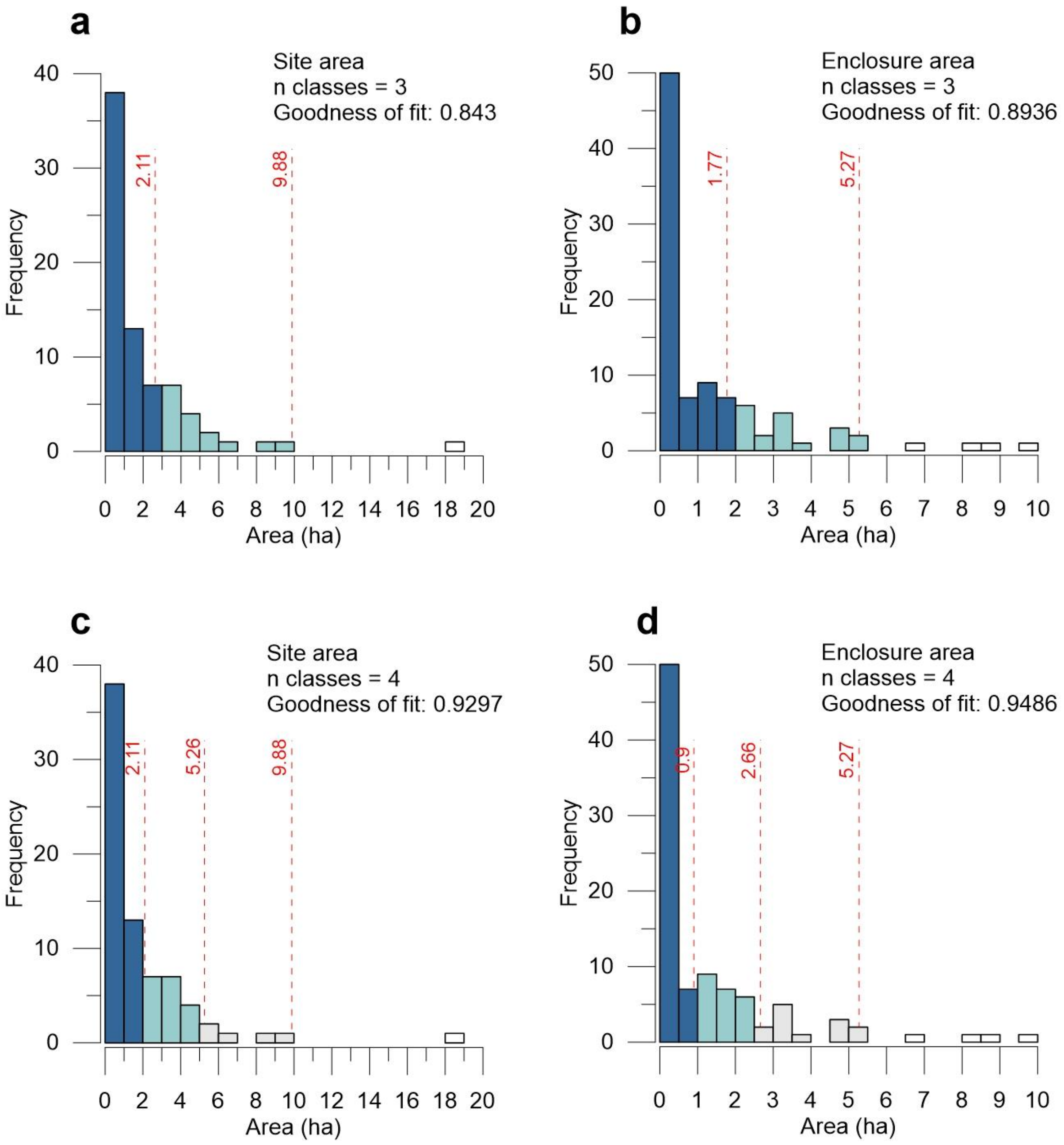

Supplementary Figure 1. Histograms of total site area and individual enclosure area in the Upper Tapajós, divided into classes using Jenks natural breaks. **a)** Three classes using total site area; **b)** Three classes using individual enclosure area; **c)** Four classes using total site area; **d)** Four classes using individual enclosure area.

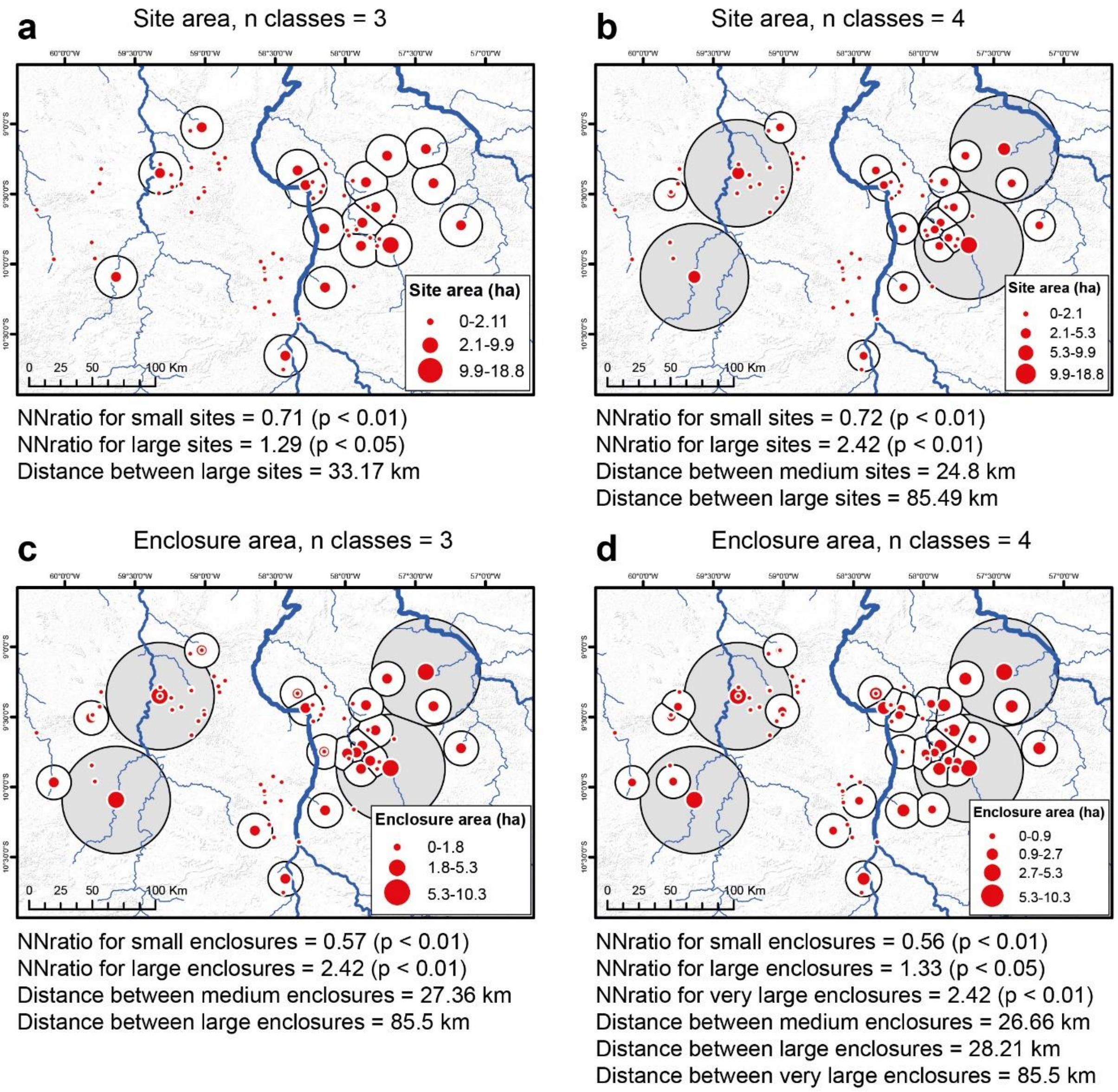

Supplementary Figure 2. Regional distribution of sites in the Upper Tapajós basin according to size class for total site area and individual enclosure area (as per Supplementary Figure 1). **a)** Three classes using total site area; **b)** Four classes using total site area; **c)** Three classes using individual enclosure area; **d)** Four classes using individual enclosure area.

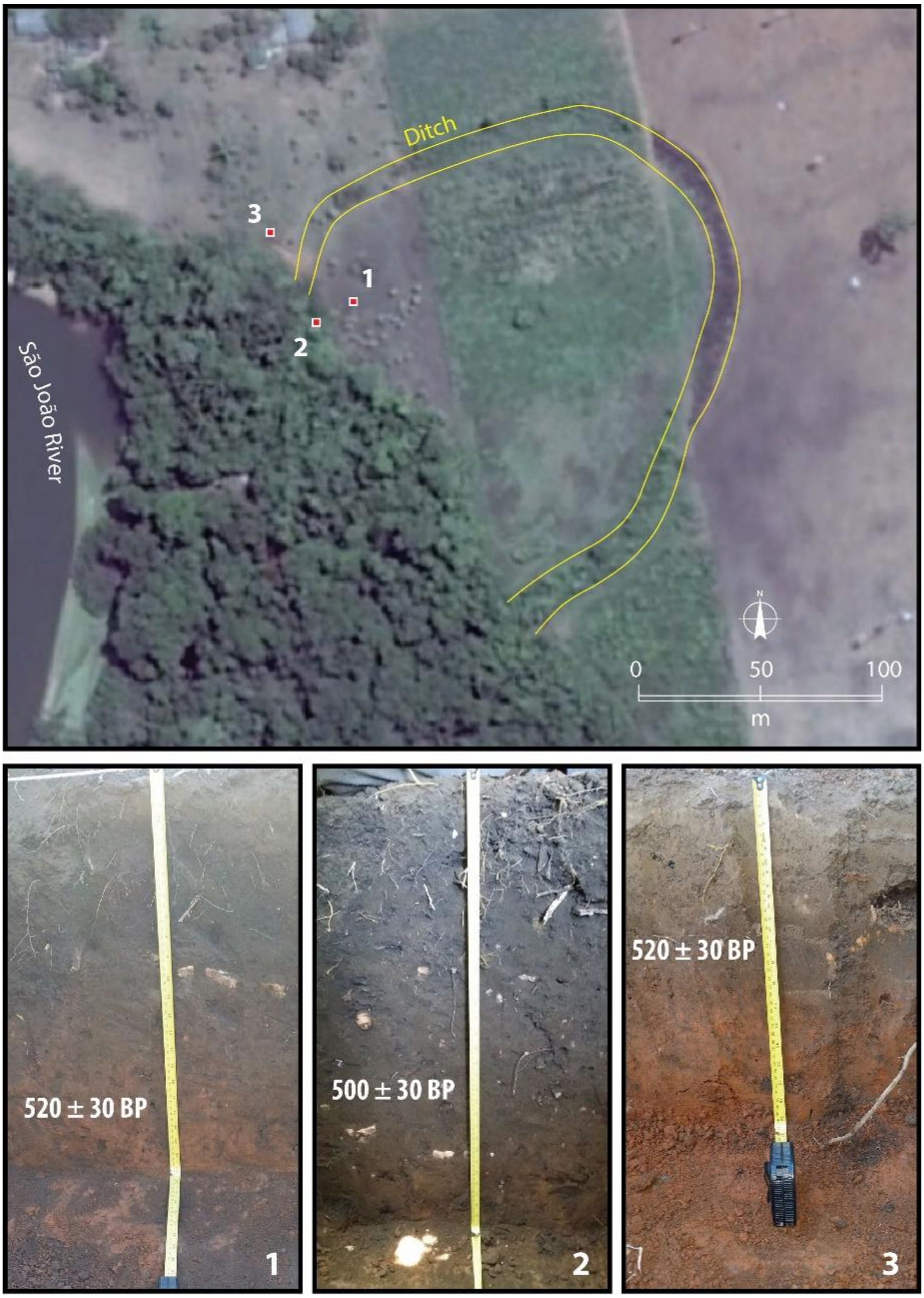

Supplementary Figure 3. Location of the excavation units at site Mt-04 (above) and position in the profile of each unit of the ^{14}C dates obtained. Notice the difference in the depth of ADE between the exterior (3) and interior (1-2) of the enclosure. Satellite image © 2017 Google, DigitalGlobe.

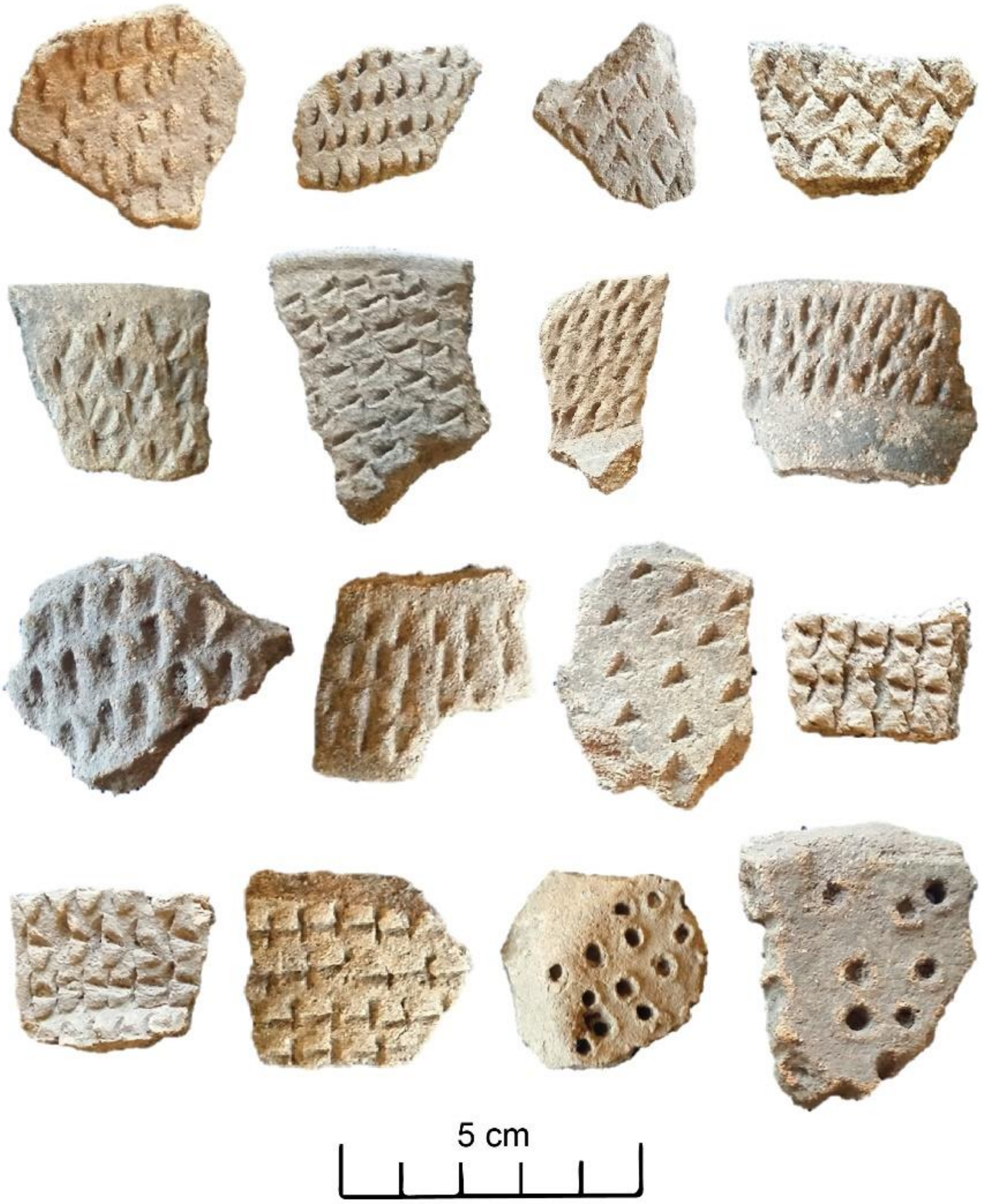

Supplementary Figure 4. A sample of surface treatments in the ceramics recovered from Mt-04, showing various nail impressions, punctations, incisions and perforated bases.

	Bio1	Bio2	Bio3	Bio4	Bio5	Bio6	Bio7	Bio8	Bio9	Bio10	Bio11	Bio12	Bio13	Bio14	Bio15	Bio16	Bio17	Bio18	Bio19
**Bio1**	1.00	-0.61	0.40	-0.41	0.28	0.82	-0.58	0.84	0.90	0.94	0.92	0.26	0.14	0.27	-0.31	0.14	0.28	-0.13	0.46
**Bio2**	-0.61	1.00	-0.66	0.44	0.50	-0.91	0.95	-0.42	-0.72	-0.53	-0.64	-0.50	-0.16	-0.64	0.67	-0.14	-0.65	-0.04	-0.67
**Bio3**	0.40	-0.66	1.00	-0.62	-0.58	0.76	-0.86	0.13	0.59	0.21	0.54	0.52	0.12	0.74	-0.71	0.11	0.74	0.15	0.63
**Bio4**	-0.41	0.44	-0.62	1.00	0.17	-0.60	0.57	0.06	-0.63	-0.09	-0.72	-0.50	-0.33	-0.33	0.35	-0.37	-0.34	0.05	-0.45
**Bio5**	0.28	0.50	-0.58	0.17	1.00	-0.24	0.58	0.32	0.10	0.37	0.16	-0.36	0.05	-0.59	0.59	0.05	-0.60	-0.35	-0.28
**Bio6**	0.82	-0.91	0.76	-0.60	-0.24	1.00	-0.93	0.54	0.92	0.70	0.87	0.49	0.20	0.59	-0.61	0.19	0.60	-0.06	0.71
**Bio7**	-0.58	0.95	-0.86	0.57	0.58	-0.93	1.00	-0.33	-0.73	-0.44	-0.67	-0.55	-0.15	-0.72	0.74	-0.14	-0.73	-0.08	-0.71
**Bio8**	0.84	-0.42	0.13	0.06	0.32	0.54	-0.33	1.00	0.56	0.92	0.60	0.02	-0.12	0.16	-0.23	-0.13	0.16	0.04	0.12
**Bio9**	0.90	-0.72	0.59	-0.63	0.10	0.92	-0.73	0.56	1.00	0.78	0.95	0.39	0.28	0.37	-0.38	0.27	0.38	-0.24	0.66
**Bio10**	0.94	-0.53	0.21	-0.09	0.37	0.70	-0.44	0.92	0.78	1.00	0.76	0.09	0.05	0.16	-0.19	0.03	0.16	-0.17	0.36
**Bio11**	0.92	-0.64	0.54	-0.72	0.16	0.87	-0.67	0.60	0.95	0.76	1.00	0.39	0.25	0.32	-0.35	0.26	0.33	-0.15	0.54
**Bio12**	0.26	-0.50	0.52	-0.50	-0.36	0.49	-0.55	0.02	0.39	0.09	0.39	1.00	0.67	0.78	-0.68	0.73	0.80	0.58	0.56
**Bio13**	0.14	-0.16	0.12	-0.33	0.05	0.20	-0.15	-0.12	0.28	0.05	0.25	0.67	1.00	0.22	0.00	0.97	0.23	0.20	0.39
**Bio14**	0.27	-0.64	0.74	-0.33	-0.59	0.59	-0.72	0.16	0.37	0.16	0.32	0.78	0.22	1.00	-0.92	0.23	0.99	0.54	0.57
**Bio15**	-0.31	0.67	-0.71	0.35	0.59	-0.61	0.74	-0.23	-0.38	-0.19	-0.35	-0.68	0.00	-0.92	1.00	-0.03	-0.94	-0.52	-0.49
**Bio16**	0.14	-0.14	0.11	-0.37	0.05	0.19	-0.14	-0.13	0.27	0.03	0.26	0.73	0.97	0.23	-0.03	1.00	0.25	0.26	0.39
**Bio17**	0.28	-0.65	0.74	-0.34	-0.60	0.60	-0.73	0.16	0.38	0.16	0.33	0.80	0.23	0.99	-0.94	0.25	1.00	0.55	0.58
**Bio18**	-0.13	-0.04	0.15	0.05	-0.35	-0.06	-0.08	0.04	-0.24	-0.17	-0.15	0.58	0.20	0.54	-0.52	0.26	0.55	1.00	-0.04
**Bio19**	0.46	-0.67	0.63	-0.45	-0.28	0.71	-0.71	0.12	0.66	0.36	0.54	0.56	0.39	0.57	-0.49	0.39	0.58	-0.04	1.00

Supplementary Figure 5. Pearson correlation matrix for the bioclimatic variables in the model.

	Base sat.	Bulk d.	Bulk ref.	Calcium c.	Gypsum	CEC clay	Clay	CEC soil	Sodicity	Salinity	Gravel	OC	pH	Sand	Silt	Exch. bases
**Base sat.**	1.00	0.31	0.26	0.43	0.54	0.66	-0.22	0.58	0.25	0.33	-0.08	-0.01	0.61	0.12	0.35	0.77
**Bulk d.**	0.31	1.00	0.80	0.06	0.05	0.15	0.13	0.13	0.05	0.22	0.10	0.09	0.79	0.50	0.17	0.09
**Bulk ref.**	0.26	0.80	1.00	0.03	0.02	0.20	0.09	0.13	0.03	0.16	0.08	0.25	0.67	0.44	0.21	0.07
**Calcium c.**	0.43	0.06	0.03	1.00	0.60	0.34	-0.02	0.30	0.84	0.64	-0.04	-0.08	0.35	-0.04	0.12	0.43
**Gypsum**	0.54	0.05	0.02	0.60	1.00	0.53	0.01	0.59	0.40	0.12	-0.06	-0.08	0.37	-0.11	0.18	0.75
**CEC clay**	0.66	0.15	0.20	0.34	0.53	1.00	-0.16	0.88	0.20	0.17	-0.18	0.12	0.40	-0.22	0.67	0.87
**Clay**	-0.22	0.13	0.09	-0.02	0.01	-0.16	1.00	0.14	-0.03	-0.07	0.09	0.28	0.20	-0.50	-0.04	-0.02
**CEC soil**	0.58	0.13	0.13	0.30	0.59	0.88	0.14	1.00	0.15	0.06	-0.20	0.15	0.38	-0.38	0.59	0.90
**Sodicity**	0.25	0.05	0.03	0.84	0.40	0.20	-0.03	0.15	1.00	0.71	-0.03	-0.06	0.23	0.02	0.03	0.22
**Salinity**	0.33	0.22	0.16	0.64	0.12	0.17	-0.07	0.06	0.71	1.00	-0.02	-0.07	0.31	0.21	-0.02	0.16
**Gravel**	-0.08	0.10	0.08	-0.04	-0.06	-0.18	0.09	-0.20	-0.03	-0.02	1.00	-0.07	0.05	0.10	-0.14	-0.13
**OC**	-0.01	0.09	0.25	-0.08	-0.08	0.12	0.28	0.15	-0.06	-0.07	-0.07	1.00	0.25	-0.11	0.17	0.01
**pH**	0.61	0.79	0.67	0.35	0.37	0.40	0.20	0.38	0.23	0.31	0.05	0.25	1.00	0.25	0.35	0.43
**Sand**	0.12	0.50	0.44	-0.04	-0.11	-0.22	-0.50	-0.38	0.02	0.21	0.10	-0.11	0.25	1.00	-0.48	-0.24
**Silt**	0.35	0.17	0.21	0.12	0.18	0.67	-0.04	0.59	0.03	-0.02	-0.14	0.17	0.35	-0.48	1.00	0.48
**Exch. bases**	0.77	0.09	0.07	0.43	0.75	0.87	-0.02	0.90	0.22	0.16	-0.13	0.01	0.43	-0.24	0.48	1.00

Supplementary Figure 6. Pearson correlation matrix for the edaphic variables in the model.

	D. Rivers	Elevation	Slope	TPI	TRI
**D. Rivers**	1.00	0.30	0.11	0.00	0.12
**Elevation**	0.30	1.00	0.48	0.00	0.45
**Slope**	0.11	0.48	1.00	0.04	0.66
**TPI**	0.00	0.00	0.04	1.00	0.01
**TRI**	0.12	0.45	0.66	0.01	1.00

Supplementary Figure 7. Pearson correlation matrix for the terrain variables in the model.

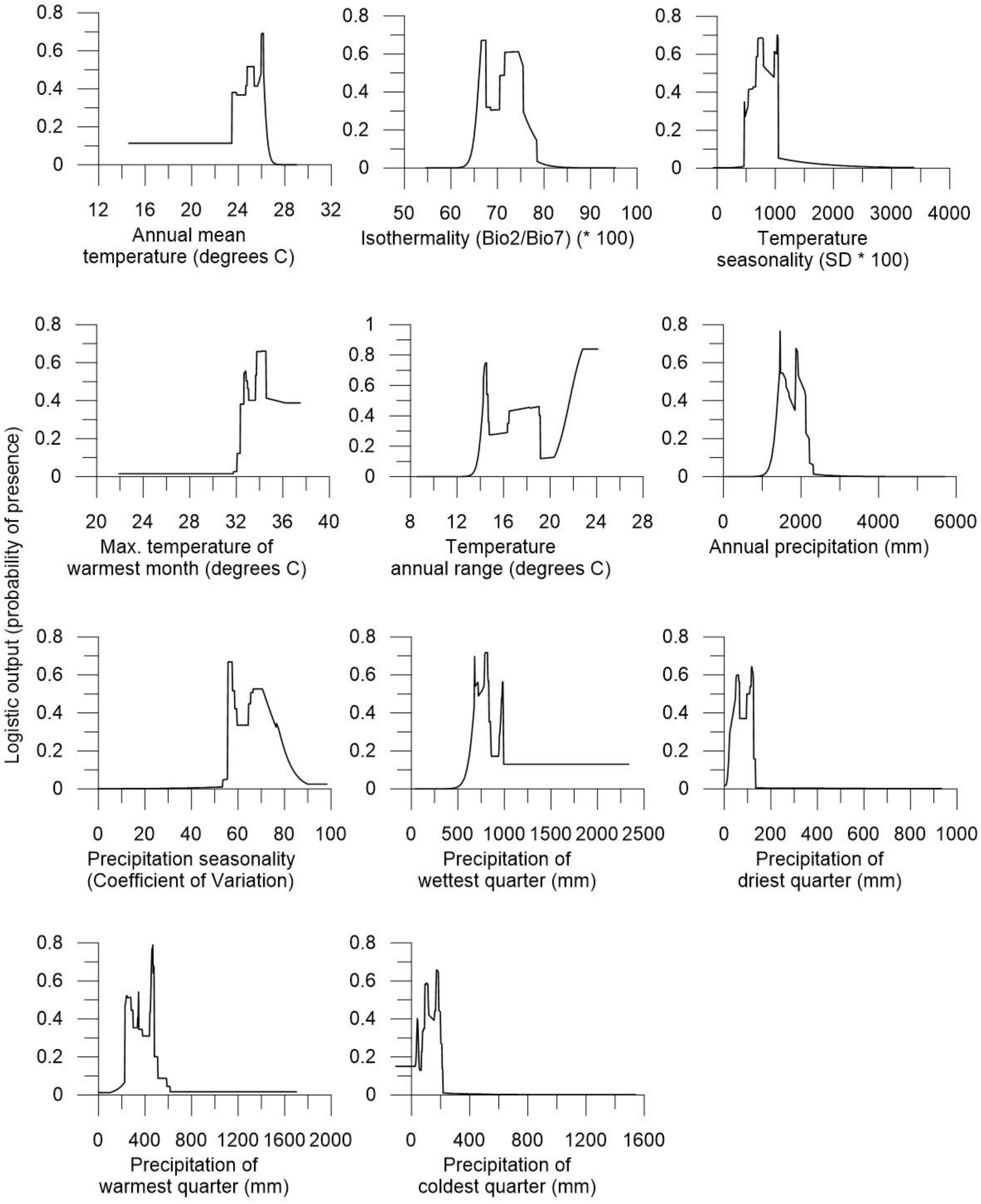

Supplementary Figure 8. Response curves for the bioclimatic variables included in the MaxEnt predictive model. Curves represent a model created using only the corresponding variable, as recommended for ease of interpretation due to the correlation between many of the variables.

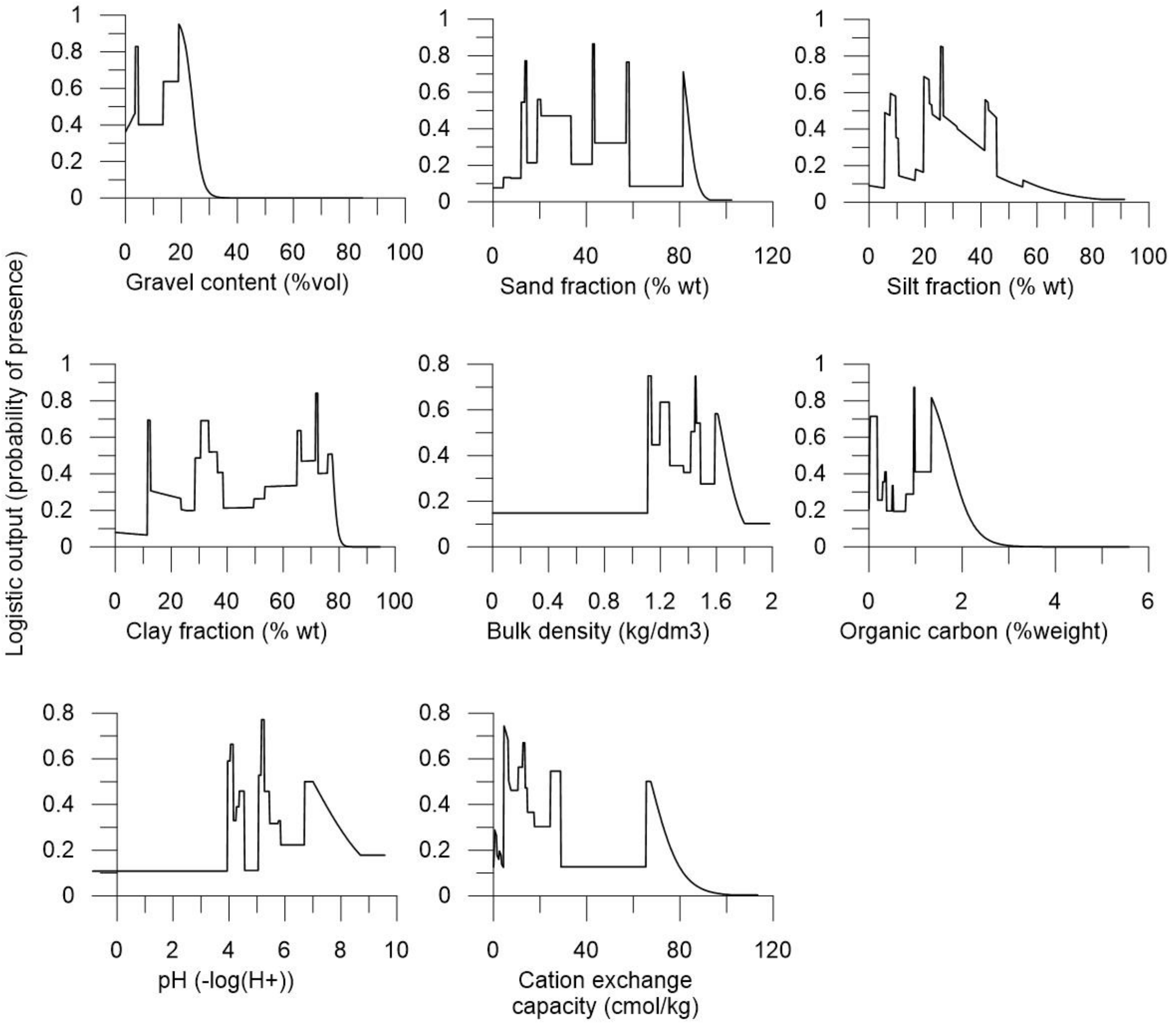

Supplementary Figure 9. Response curves for the edaphic variables included in the MaxEnt predictive model. Curves represent a model created using only the corresponding variable, as recommended for ease of interpretation due to the correlation between many of the variables.

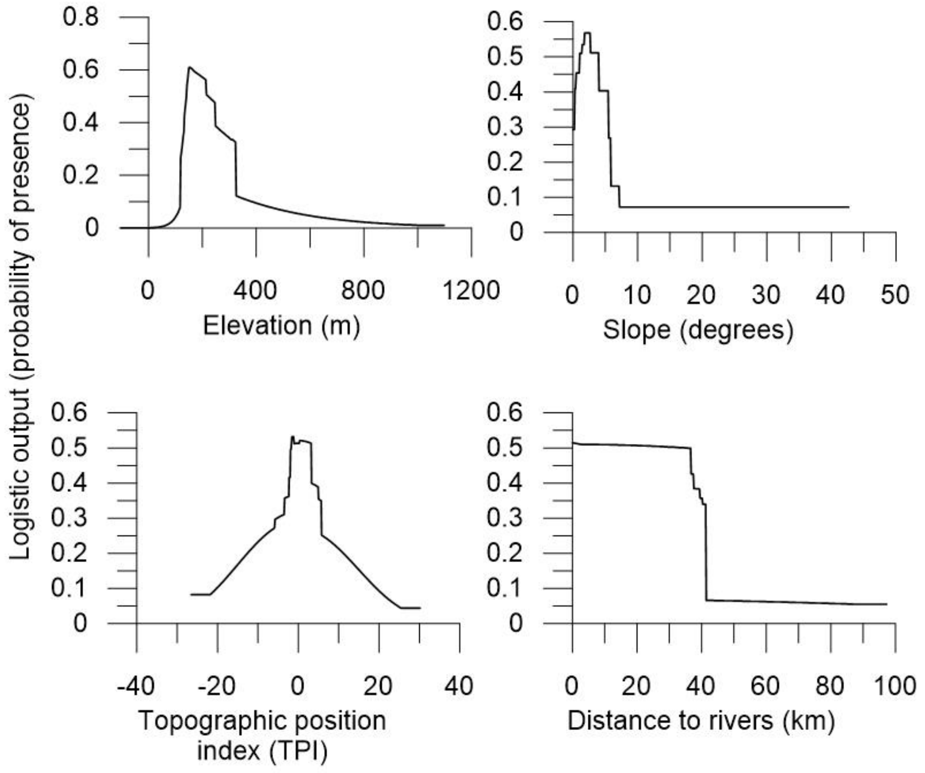

Supplementary Figure 10. Response curves for the terrain variables included in the MaxEnt predictive model. Curves represent a model created using only the corresponding variable, as recommended for ease of interpretation due to the correlation between many of the variables.

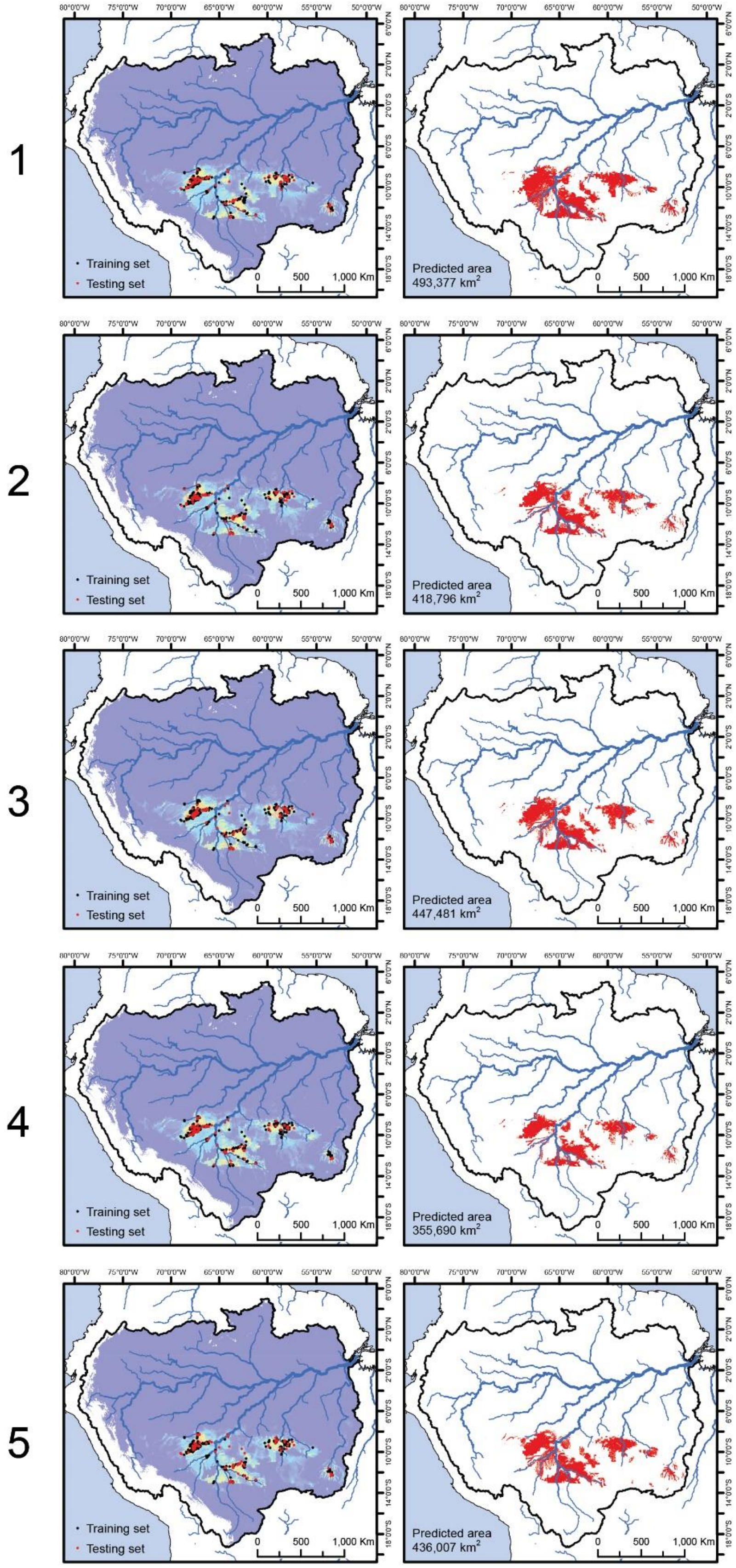

Supplementary Figure 11. Ten repetitions of the MaxEnt model using a random 25% of archaeological sites as testing samples (left) together with the predicted total area of

earthwork occurrence using maximum training sensitivity plus specificity as a threshold (right).

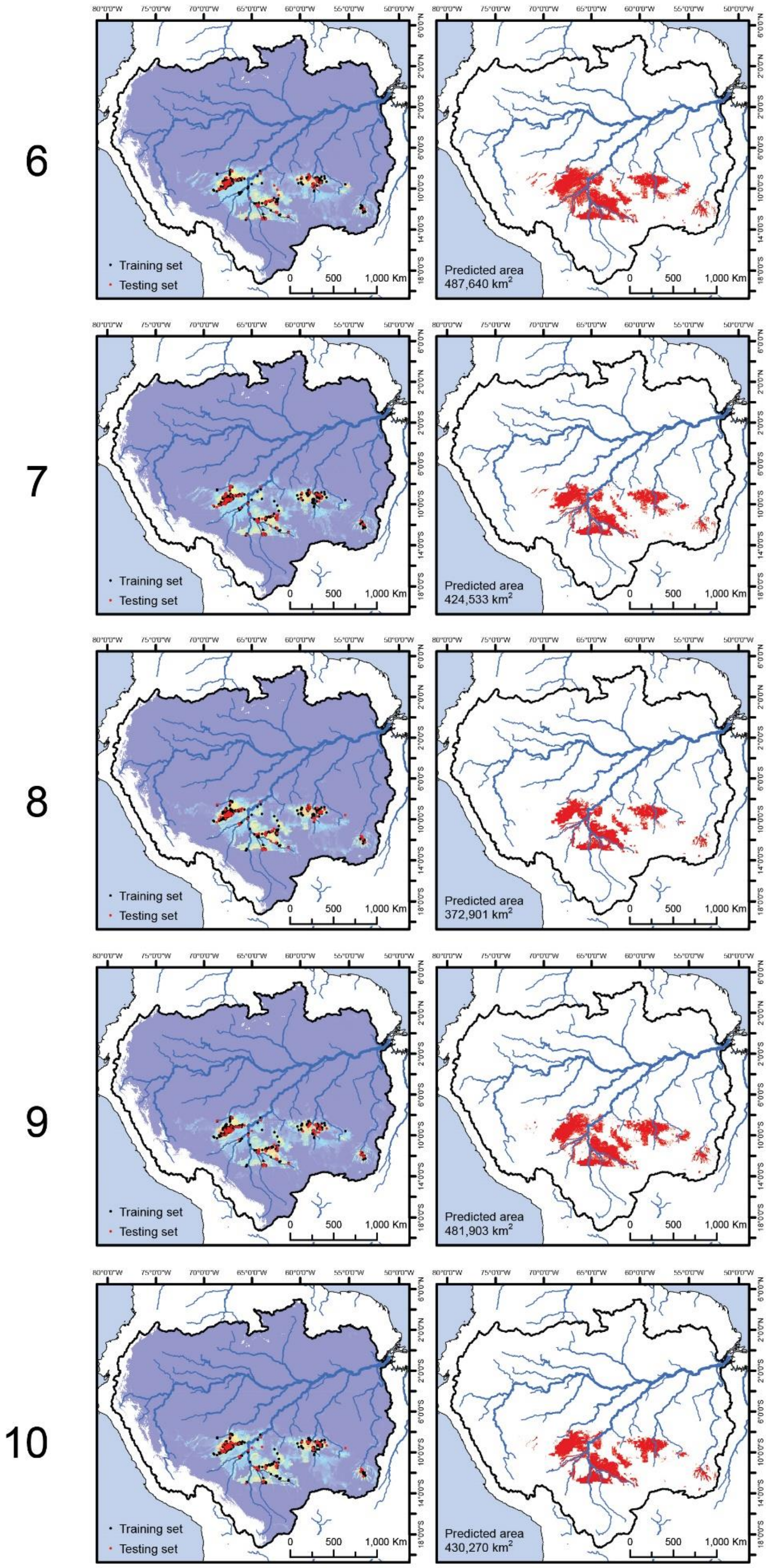

Supplementary Figure 11 (cont.). Ten repetitions of the MaxEnt model using a random 25% of archaeological sites as testing samples (left) together with the predicted total area of earthwork occurrence using maximum training sensitivity plus specificity as a threshold (right).

Supplementary References

1 Pärssinen, M., Schaan, D. P. & Ranzi, A. Pre-Columbian geometric earthworks in the upper Purús: a complex society in western Amazonia. *Antiquity* **83**, 1084-1095 (2009).

2 Schaan, D. *et al.* New radiometric dates for precolumbian (2000-700 B.P.) earthworks in western Amazonia, Brazil. *Journal of Field Archaeology* **37**, 132-142 (2012).

3 Schaan, D. *et al.* Construindo paisagens como espaços sociais: o caso dos geoglifos do Acre. *Revista de Arqueologia* **23**, 30-41 (2010).

4 Schaan, D. P. *Sacred Geographies of Ancient Amazonia: Historical Ecology of Social Complexity*. (Left Coast Press, 2011).

5 Watling, J. *et al.* Impact of pre-Columbian "geoglyph" builders on Amazonian forests. *Proceedings of the National Academy of Sciences* **114**, 1868-1873 (2017).

6 McMichael, C. H., Palace, M. W. & Golightly, M. Bamboo-dominated forests and pre-Columbian earthwork formations in south-western Amazonia. *Journal of Biogeography* **41**, 1733-1745, doi:10.1111/jbi.12325 (2014).

7 Saunaluoma, S. & Schaan, D. Monumentality in Western Amazonian formative societies: geometric ditched enclosures in the Brazilian state of Acre. *Antiqua* **2**, 1 (2012).

8 Walker, J. H. Pre-Columbian ring ditches along the Yacuma and Rapulo Rivers, Beni, Bolivia: A preliminary review. *Journal of Field Archaeology* **33**, 413-427 (2008).

9 Erickson, C. L., Álvarez, P. & Calla, S. Zanjas circundantes: obras de tierra monumentales de Baures en la Amazonia Boliviana. (Unidad Nacional de Arqueología, La Paz, 2008).

10 Saunaluoma, S. Pre-Columbian earthworks in the Riberalta region of the Bolivian Amazon. *Amazônica-Revista de Antropologia* **2** (2010).

11 Carson, J. F. *et al.* Environmental impact of geometric earthwork construction in pre-Columbian Amazonia. *Proceedings of the National Academy of Sciences* **111**, 10497-10502 (2014).

12 Prümers, H. & Jaimes Betancourt, C. 100 años de investigación arqueológica en los Llanos de Mojos. *Arqueoantropológicas* **4**, 11-54 (2014).

13 Eder, F. J. *Breve descripción de las reducciones de Mojos*. (J. Barnadas, 1985).

14 Heckenberger, M. J. *et al.* Pre-Columbian Urbanism, Anthropogenic Landscapes, and the Future of the Amazon. *Science* **321**, 1214-1217 (2008).

15 Heckenberger, M. J. *et al.* Amazonia 1492: Pristine Forest or Cultural Parkland? *Science* **301**, 1710-1714 (2003).

16 Neves, E. G. *et al.* Pesquisa e Formação nos Sítios Arqueológicos Espinhara e Sol de Campinas do Acre - PESC. (University of São Paulo, São Paulo, 2016).

17 Wüst, I. & Barreto, C. The ring villages of central Brazil: a challenge for Amazonian archaeology. *Latin American Antiquity* **10**, 3-23 (1999).

18 Lévi-Strauss, C. in *Anthropologie Structurale* (ed Claude Lévi-Strauss) 147-180 (Plon, 1958).

19 Hornborg, A. Ethnogenesis, Regional Integration, and Ecology in Prehistoric Amazonia: Toward a System Perspective. *Current Anthropology* **46**, 589-620 (2005).

20 Heckenberger, M. J. *The ecology of power: culture, place, and personhood in the southern Amazon, A.D. 1000-2000*. (Routledge, 2005).

21 Hill, J. D. & Santos-Granero, F. *Comparative Arawakan histories: rethinking language family and culture area in Amazonia*. (University of Illinois Press, 2002).

22 Simões, M. F. Pesquisa e cadastro de sítios arqueológicos na Amazônia Legal brasileira 1978-1982. *Publicações Avulsas do Museu Paraense Emílio Goeldi* **38**, 57-60 (1983).

23 Prümers, H. in *Amazonía: Memorias de las Conferencias Magistrales del 3er Encuentro Internacional de Arqueología Amazónica* (ed Stéphen Rostain) 73-89 (Ekseption Publicidad, 2014).

24 Curet, L. A. New formulae for estimating prehistoric populations for lowland South America and the Caribbean. *Antiquity* **72**, 359-375 (1998).
